# Supplementary material for: Isolation and Characterization of Phages Active against Paenibacillus larvae Causing American Foulbrood in Honeybees in Poland
Source: Viruses. 2021 Jun 23;13(7):1217. doi: 10.3390/v13071217 (PMC8310151; doi:10.3390/v13071217)
Supplement: Supplementary file 1 [file viruses-13-01217-s001.zip › viruses-1226363-supplementary.pdf]

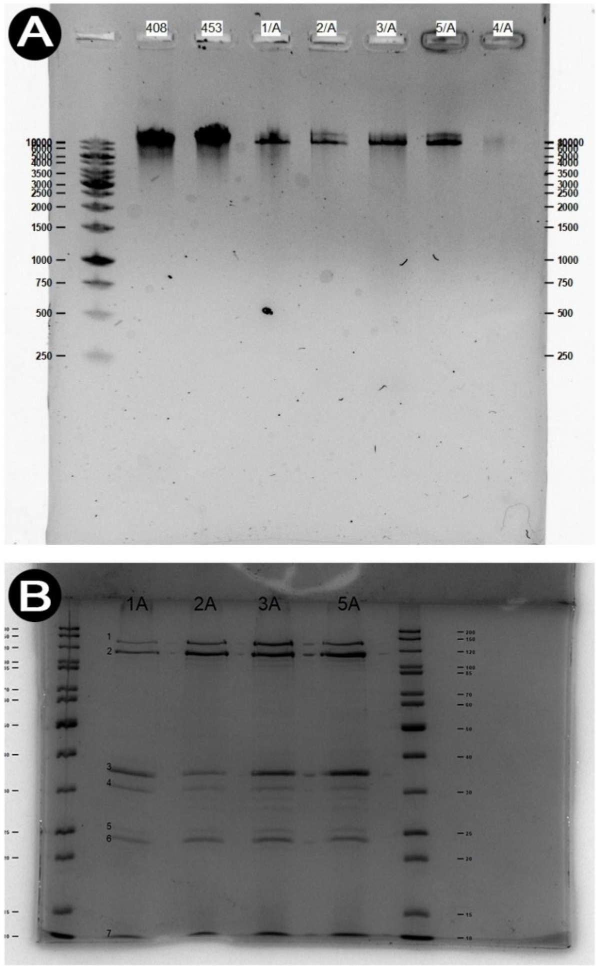

Figure S1 A) DNA isolated from *Paenibacillus larvae* 408, 453 and phages 1A-5/A, B) Protein profiles of 1/A-5/A bacteriophages

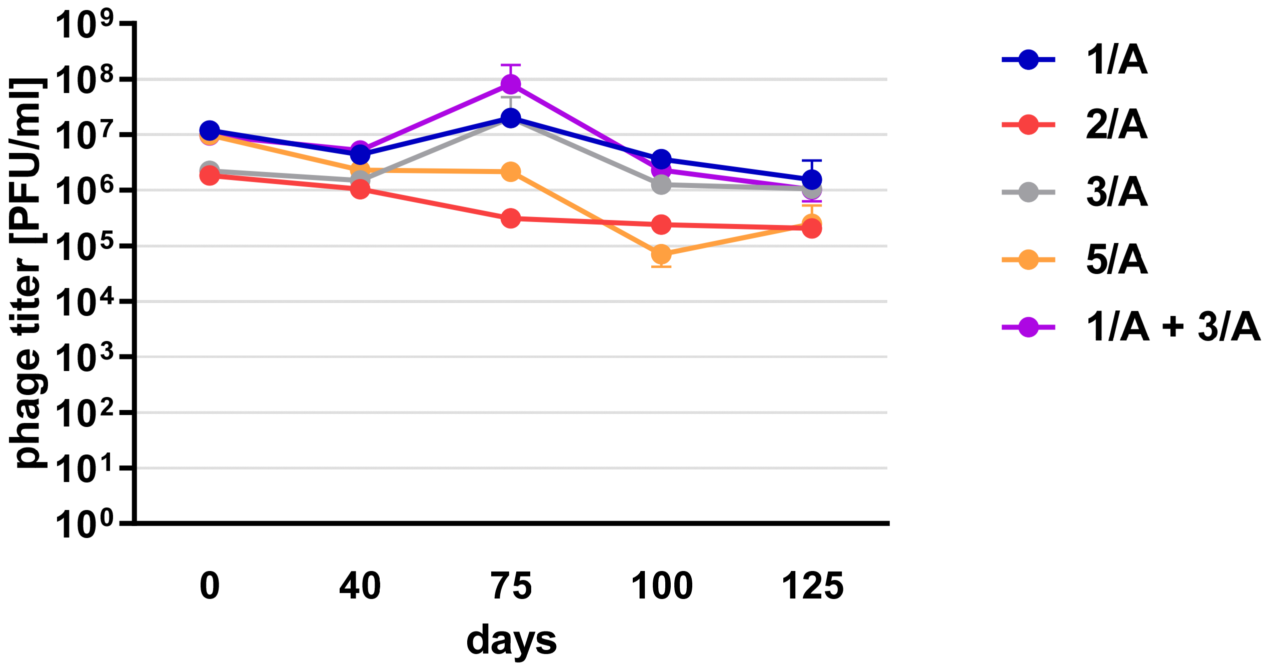

Figure S2. The stability of lyophilized phages with 10% glucose. Error bars represent standard deviation (±SD) of mean phage titer.

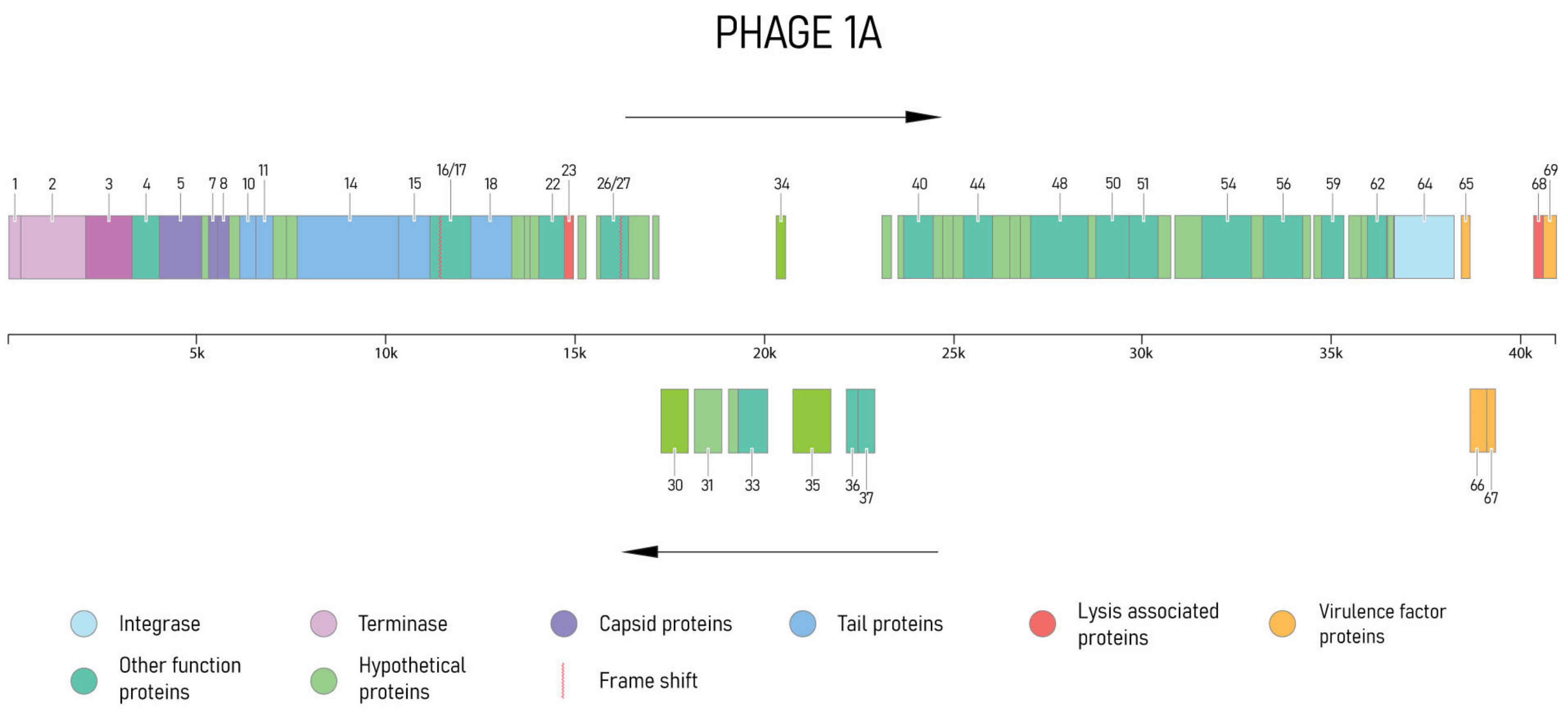
PHAGE 1A
1
2
3
4
5
7
8
10
11
14
15
16/17
18
22
23
26/27
34
40
44
48
50
51
54
56
59
62
64
65
68
69
5k
10k
15k
20k
25k
30k
35k
40k
30
31
33
35
36
37
66
67
Integrase
Terminase
Capsid proteins
Tail proteins
Lysis associated proteins
Virulence factor proteins
Other function proteins
Hypothetical proteins
Frame shift

PHAGE 2A

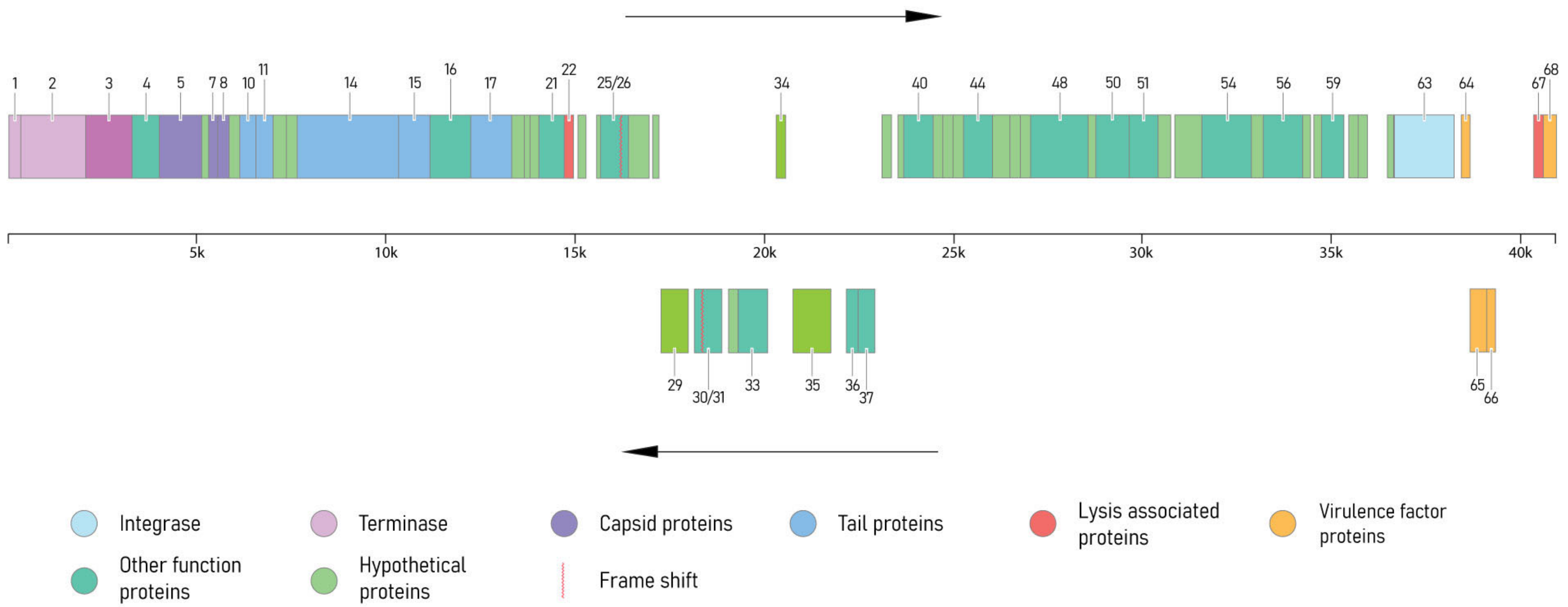

1
2
3
4
5
7
8
10
11
14
15
16
17
21
22
25/26
34
40
44
48
50
51
54
56
59
63
64
67
68
5k
10k
15k
20k
25k
30k
35k
40k
29
30/31
33
35
36
37
65
66
Integrase
Terminase
Capsid proteins
Tail proteins
Lysis associated proteins
Virulence factor proteins
Other function proteins
Hypothetical proteins
Frame shift

PHAGE 3A

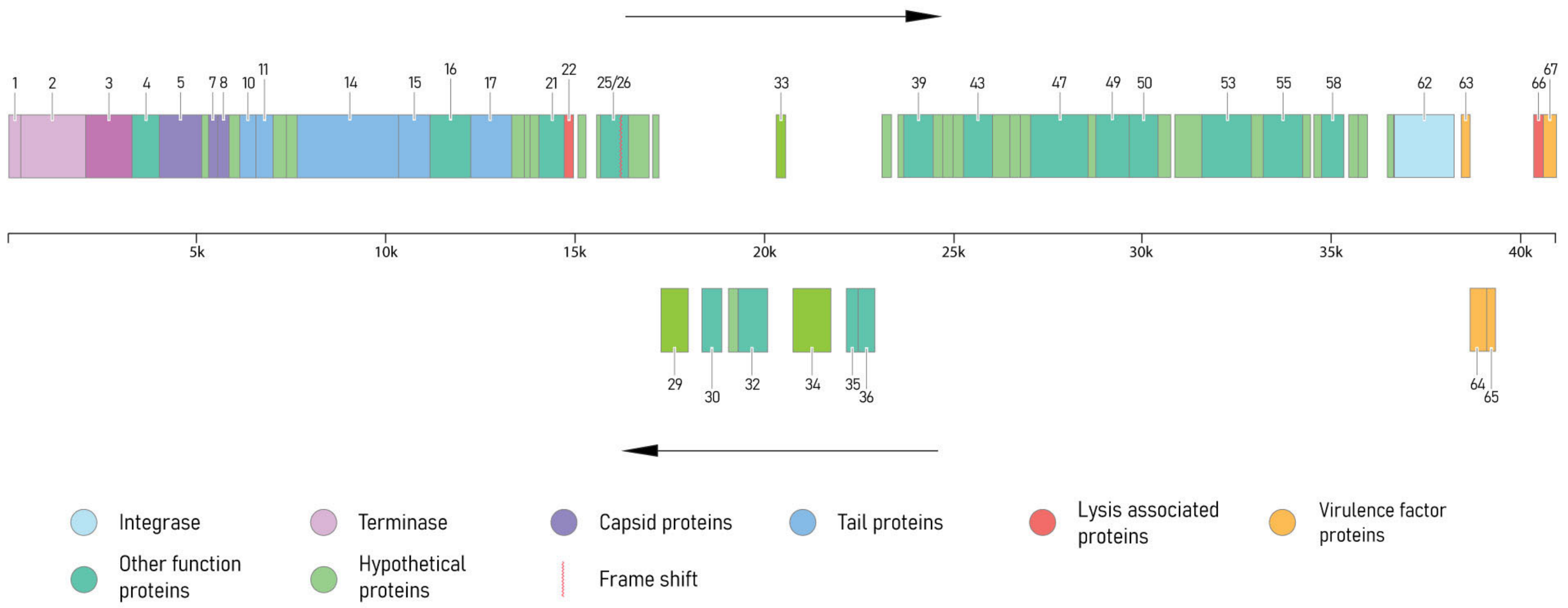
1
2
3
4
5
7
8
10
11
14
15
16
17
21
22
25/26
33
39
43
47
49
50
53
55
58
62
63
66
67
5k
10k
15k
20k
25k
30k
35k
40k
29
30
32
34
35
36
64
65
Integrase
Terminase
Capsid proteins
Tail proteins
Lysis associated proteins
Virulence factor proteins
Other function proteins
Hypothetical proteins
Frame shift

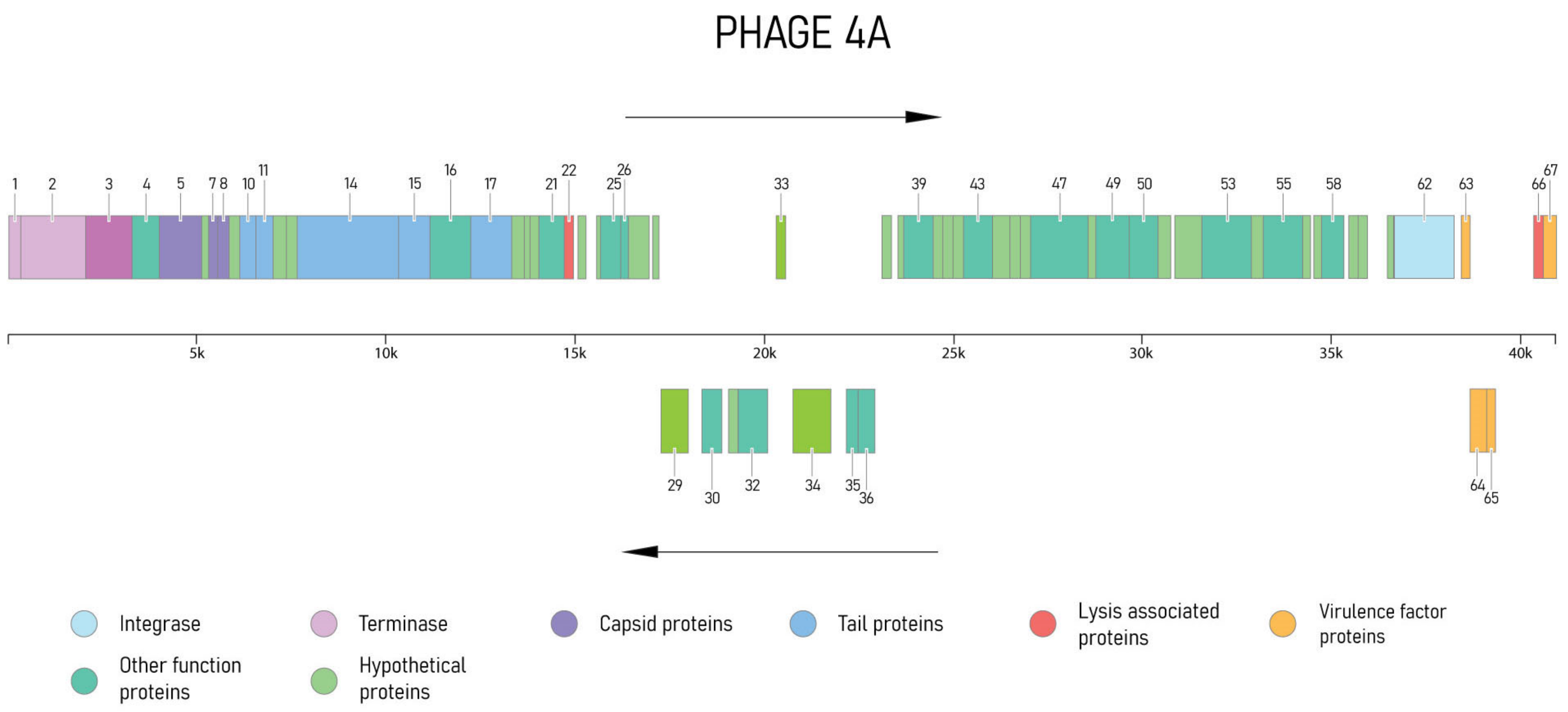
PHAGE 4A
1
2
3
4
5
7
8
10
11
14
15
16
17
21
22
25
26
33
39
43
47
49
50
53
55
58
62
63
66
67
5k
10k
15k
20k
25k
30k
35k
40k
29
30
32
34
35
36
64
65
Integrase
Terminase
Capsid proteins
Tail proteins
Lysis associated proteins
Virulence factor proteins
Other function proteins
Hypothetical proteins

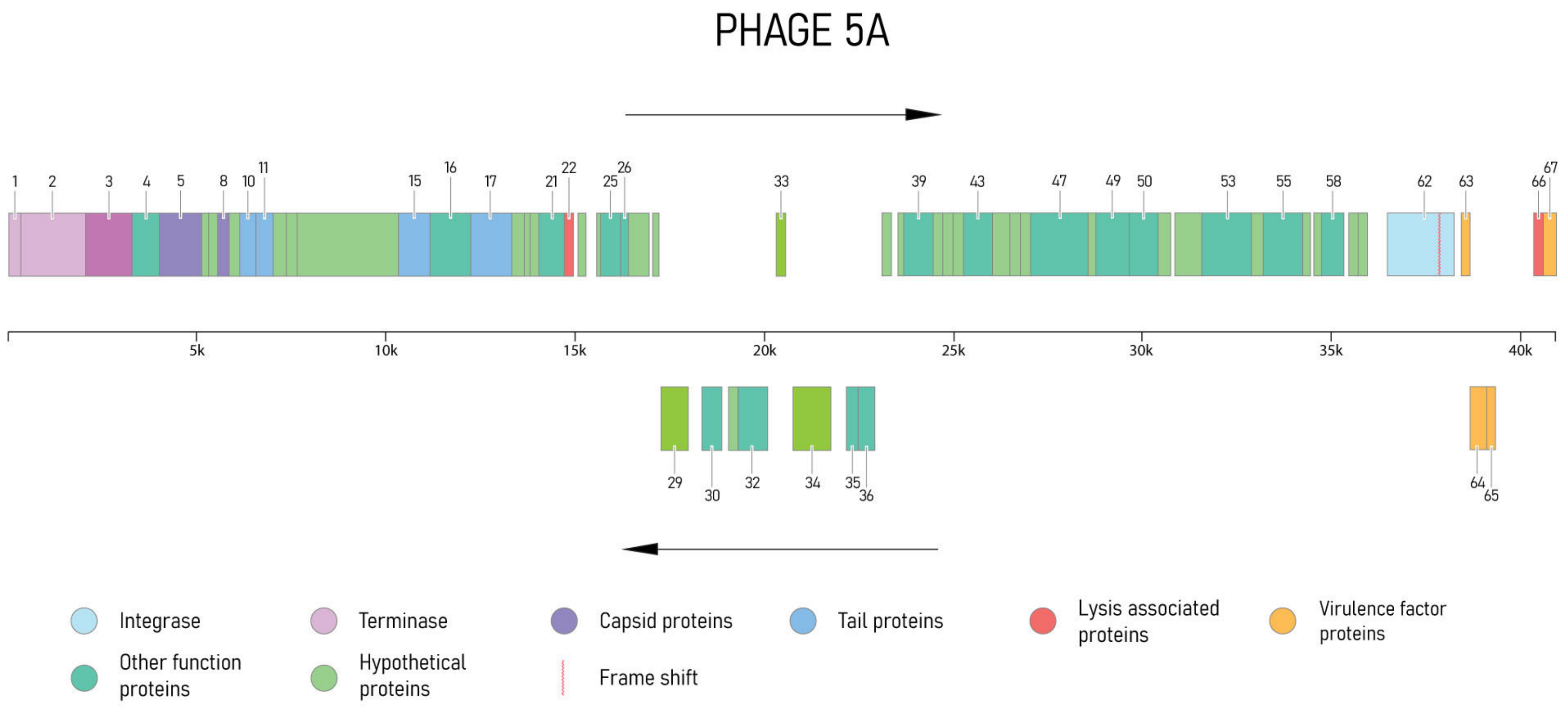

Figure S3. Genomics maps of studied phages 1-5/A

Table S1. Annotation table of phage 1/A

#	Location		Strand	Size		Probable function	Homologue	Comments
	Start	End		nt	aa			
1	45	359	+	315	104	terminase, small subunit	YP_009195191.1	-
2	340	2064	+	1725	574	terminase, large subunit	YP_008320338.1	-
3	2061	3314	+	1254	417	portal protein	YP_009203205.1	-
4	3298	4020	+	723	240	Clp, protease	YP_009203206.1	-
5	4017	5147	+	1131	376	major capsid protein	YP_008320341.1	-
6	5125	5298	+	174	57	hypothetical protein	YP_008320342.1	-
7	5291	5554	+	264	87	head-tail connector protein	YP_008320343.1	-
8	5551	5868	+	318	105	head closure protein	YP_008320344.1	-
9	5868	6128	+	261	86	hypothetical protein	YP_009195199.1	-
10	6138	6578	+	441	146	tail protein	YP_008320346.1	-
11	6580	7017	+	438	145	major tail protein	YP_008320347.1	-
12	7014	7352	+	339	112	hypothetical protein	YP_009195202.1	-
13	7379	7633	+	255	84	hypothetical protein	YP_008320349.1	-
14	7693	10329	+	2637	878	tail tape measure protein	YP_008320350.1	-
15	10326	11177	+	852	283	tail protein	YP_008320351.1	-
16	11180	11440	+	261	86	*Siphovirus* ReqiPepy6 Gp37-like protein	YP_008320352.1	Potential reading frame shift.
17	11445	12239	+	795	264	*Siphovirus* ReqiPepy6 Gp37-like protein	YP_008320352.1	
18	12236	13318	+	1083	360	tail protein	YP_009201927.1	-
19	13319	13666	+	348	115	hypothetical protein	YP_009201928.1	-
20	13663	13824	+	162	53	hypothetical protein	YP_009197964.1	-

21	13805	14044	+	240	79	hypothetical protein	YP_009197965.1	-
22	14041	14715	+	675	224	N-acetylmuramoyl-L-alanine amidase	YP_009201931.1	-
23	14725	14967	+	243	80	holin	YP_009197967.1	-
24	15101	15292	+	192	63	hypothetical protein	YP_009197968.1	-
25	15574	15684	+	111	36	hypothetical protein	YP_009197969.1	-
26	15681	16202	+	522	173	amidase domain-containing protein	YP_009197970.1	Potential reading frame shift.
27	16199	16387	+	189	62	amidase domain-containing protein	YP_009197970.1	
28	16423	16947	+	525	174	hypothetical protein	YP_009197971.1	-
29	17111	17218	+	108	35	hypothetical protein	YP_009197972.1	-
30	17280	17999	-	720	239	transposase	YP_009201939.1	-
31	18170	18853	-	684	227	helix-turn-helix domain-containing protein	YP_009197974.1	-
32	19339	19542	-	204	67	hypothetical protein	YP_009197976.1	-
33	19544	20092	-	549	182	YolD-like family protein	YP_009197977.1	-
34	20334	20588	+	255	84	transposase	YP_009197978.1	-
35	20786	21475	-	690	229	transposase	YP_009197979.1	-
z	21481	21762	-	282	93	helix-turn-helix transcriptional regulator	YP_009197980.1	-
37	22472	22933	-	462	153	helix-turn-helix domain-containing protein	YP_009197981.1	-
38	23134	23391	+	258	85	hypothetical protein	YP_009201956.1	-
39	23537	23692	+	156	51	hypothetical protein	YP_009201956.1	-
40	23718	24458	+	741	246	antirepressor	YP_009197983.1	-
41	24485	24703	+	219	72	hypothetical protein	YP_009201958.1	-
42	24727	24996	+	270	89	hypothetical protein	YP_009201959.1	-
43	24993	25265	+	273	90	hypothetical protein	YP_009201960.1	-

44	25292	26050	+	759	252	antirepressor	YP_009201961.1	-
45	26047	26526	+	480	159	hypothetical protein	YP_009201962.1	-
46	26516	26764	+	249	82	hypothetical protein	YP_009201963.1	-
47	26769	27059	+	291	96	hypothetical protein	YP_009197985.1	-
48	27043	28575	+	1533	510	ATPase	YP_009197986.1	-
49	28562	28759	+	198	65	hypothetical protein	YP_009197987.1	-
50	28762	29664	+	903	300	recombinase RecT	YP_009197988.1	-
51	29677	30435	+	759	252	MBL fold metallo-hydrolase	YP_009197989.1	-
52	30432	30746	+	315	104	hypothetical protein	YP_008320387.1	-
53	30760	31590	+	831	276	hypothetical protein	YP_008320388.1	-
54	31556	32878	+	1323	440	DNA helicase	YP_009197992.1	-
55	32875	33186	+	312	103	hypothetical protein	YP_009201972.1	-
56	33179	34234	+	1056	351	DNA (cytosine-5-)-methyltransferase	YP_009201973.1	-
57	34221	34430	+	210	69	hypothetical protein	YP_009201974.1	-
58	34515	34685	-	171	56	hypothetical protein	-	-
59	34721	35311	+	591	196	resolvase	YP_009838694.1	-
60	35470	35775	+	306	101	hypothetical protein	YP_009201977.1	-
61	35787	35936	+	150	49	hypothetical protein	YP_009838964.1	-
62	35987	36445	+	459	152	transcriptional regulator	YP_009201978.1	-
63	36457	36738	+	282	93	hypothetical protein	YP_009201979.1	-
64	36677	38239	+	1563	520	integrase	YP_009201980.1	-
65	38379	38654	+	276	91	phosphomannomutase	YP_009201981.1	-
66	38683	39093	-	411	136	type II toxin-antitoxin system HicB family antitoxin	YP_009201982.1	-

67	39124	39360	-	237	78	type II toxin-antitoxin system HicA family toxin	YP_009201983.1	-
68	40337	40603	+	267	88	transglycosylase	YP_009201986.1	-
69	40600	40938	+	339	112	HNH endonuclease	YP_009201987.1	-

Table S2. Annotation table of phage 2/A

#	Location		Strand	Size		Probable function	Homologue	Comments
	Start	End		nt	aa			
1	2	304	+	303	100	terminase, small subunit	YP_009195191.1	-
2	285	2009	+	1725	574	terminase, large subunit	YP_008320338.1	-
3	2006	3259	+	1254	417	portal protein	YP_009203205.1	-
4	3243	3965	+	723	240	Clp, protease	YP_009203206.1	-
5	3962	5092	+	1131	376	major capsid protein	YP_008320341.1	-
6	5070	5243	+	174	57	hypothetical protein	YP_008320342.1	-
7	5236	5499	+	264	87	head-tail connector protein	YP_008320343.1	-
8	5496	5813	+	318	105	head closure protein	YP_008320344.1	-
9	5813	6241	+	429	142	hypothetical protein	YP_008320345.1	-
10	6225	6620	+	396	131	tail protein	YP_008320346.1	-
11	6622	7059	+	438	145	major tail protein	YP_008320347.1	-
12	7056	7394	+	339	112	hypothetical protein	YP_009195202.1	-
13	7421	7675	+	255	84	hypothetical protein	YP_008320349.1	-
14	7735	10371	+	2637	878	tail tape measure protein	YP_008320350.1	-
15	10368	11219	+	852	283	tail protein	YP_008320351.1	-

16	11222	12340	+	1119	372	*Siphovirus* ReqiPepy6 Gp37-like protein	YP_008320352.1	-
17	12337	13419	+	1083	360	tail protein	YP_009201927.1	-
18	13420	13767	+	348	115	hypothetical protein	YP_009201928.1	-
19	13764	13925	+	162	53	hypothetical protein	YP_009197964.1	-
20	13906	14145	+	240	79	hypothetical protein	YP_009197965.1	-
21	14142	14816	+	675	224	N-acetylmuramoyl-L-alanine amidase	YP_009201931.1	-
22	14826	15068	+	243	80	holin	YP_009197967.1	-
23	15202	15393	+	192	63	hypothetical protein	YP_009197968.1	-
24	15675	15785	+	111	36	hypothetical protein	YP_009197969.1	-
25	15782	16303	+	522	173	amidase domain-containing protein	YP_009197970.1	-
26	16300	16488	+	189	62	amidase domain-containing protein	YP_009197970.1	-
27	16524	17048	+	525	174	hypothetical protein	YP_009197971.1	-
28	17212	17319	+	108	35	hypothetical protein	YP_009197972.1	-
29	17381	18100	-	720	239	transposase	YP_009201939.1	-
30	18271	18438	-	168	55	helix-turn-helix domain-containing protein	YP_009197974.1	Potential reading frame shift.
31	18454	18987	-	534	177	helix-turn-helix domain-containing protein	YP_009197974.1	
32	19473	19676	-	204	67	hypothetical protein	YP_009197976.1	-
33	19678	20226	-	549	182	YolD-like family protein	YP_009197977.1	-
34	20468	20722	+	255	84	transposase	YP_009197978.1	-
35	20920	21609	-	690	229	transposase	YP_009197979.1	-
36	21615	21896	-	282	93	helix-turn-helix transcriptional regulator	YP_009197980.1	-

37	22606	23067	-	462	153	helix-turn-helix domain-containing protein	YP_009197981.1	-
38	23268	23525	+	258	85	hypothetical protein	YP_009201956.1	-
39	23671	23826	+	156	51	hypothetical protein	YP_009201956.1	-
40	23852	24592	+	741	246	antirepressor	YP_009197983.1	-
41	24619	24837	+	219	72	hypothetical protein	YP_009201958.1	-
42	24861	25130	+	270	89	hypothetical protein	YP_009201959.1	-
43	25127	25399	+	273	90	hypothetical protein	YP_009201960.1	-
44	25426	26184	+	759	252	antirepressor	YP_009201961.1	-
45	26181	26660	+	480	159	hypothetical protein	YP_009201962.1	-
46	26650	26898	+	249	82	hypothetical protein	YP_009201963.1	-
47	26903	27193	+	291	96	hypothetical protein	YP_009197985.1	-
48	27177	28709	+	1533	510	ATPase	YP_009197986.1	-
49	28696	28893	+	198	65	hypothetical protein	YP_009197987.1	-
50	28896	29798	+	903	300	recombinase RecT	YP_009197988.1	-
51	29811	30569	+	759	252	MBL fold metallo-hydrolase	YP_009197989.1	-
52	30566	30880	+	315	104	hypothetical protein	YP_008320387.1	-
53	30894	31724	+	831	276	hypothetical protein	YP_008320388.1	-
54	31690	33012	+	1323	440	DNA helicase	YP_009197992.1	-
55	33009	33320	+	312	103	hypothetical protein	YP_009201972.1	-
56	33313	34368	+	1056	351	DNA (cytosine-5-)-methyltransferase	YP_009201973.1	-
57	34355	34564	+	210	69	hypothetical protein	YP_009201974.1	-
58	34649	34819	-	171	56	hypothetical protein	-	-
59	34855	35445	+	591	196	resolvase	YP_009838694.1	-
60	35604	36062	+	459	152	hypothetical protein	YP_009201977.1	-
61	36113	36571	+	459	152	hypothetical protein	YP_009201978.1	-

62	36583	36864	+	282	93	hypothetical protein	YP_009201979.1	-
63	36803	38365	+	1563	520	integrase	YP_009201980.1	-
64	38505	38780	+	276	91	phosphomannomutase	YP_009201981.1	-
65	38809	39219	-	411	136	type II toxin-antitoxin system HicB family antitoxin	YP_009201982.1	-
66	39250	39486	-	237	78	type II toxin-antitoxin system HicA family toxin	YP_009201983.1	-
67	40462	40728	+	267	88	transglycosylase	YP_009201986.1	-
68	40725	41063	+	339	112	HNH endonuclease	YP_009201987.1	-

Table S3. Annotation table of phage 3/A

#	Location		Strand	Size		Probable function	Homologue	Comments
	**Start**	**End**		**nt**	**aa**			
1	1	303	+	303	100	terminase, small subunit	YP_009195191.1	-
2	284	2008	+	1725	574	terminase, large subunit	YP_008320338.1	-
3	2005	3258	+	1254	417	portal protein	YP_009203205.1	-
4	3242	3964	+	723	240	Clp, protease	YP_009203206.1	-
5	3961	5091	+	1131	376	major capsid protein	YP_008320341.1	-
6	5069	5242	+	174	57	hypothetical protein	YP_008320342.1	-
7	5235	5498	+	264	87	head-tail connector protein	YP_008320343.1	-
8	5495	5812	+	318	105	head closure protein	YP_008320344.1	-
9	5812	6240	+	429	142	hypothetical protein	YP_008320345.1	-
10	6224	6619	+	396	131	tail protein	YP_008320346.1	-
11	6621	7058	+	438	145	major tail protein	YP_008320347.1	-

12	7055	7393	+	339	112	hypothetical protein	YP_009195202.1	-
13	7420	7674	+	255	84	hypothetical protein	YP_008320349.1	-
14	7734	10370	+	2637	878	tail tape measure protein	YP_008320350.1	-
15	10367	11218	+	852	283	tail protein	YP_008320351.1	-
16	11221	12339	+	1119	372	*Siphovirus* ReqiPepy6 Gp37-like protein	YP_008320352.1	-
17	12336	13418	+	1083	360	tail protein	YP_009201927.1	-
18	13419	13766	+	348	115	hypothetical protein	YP_009201928.1	-
19	13763	13924	+	162	53	hypothetical protein	YP_009197964.1	-
20	13905	14144	+	240	79	hypothetical protein	YP_009197965.1	-
21	14141	14815	+	675	224	N-acetylmuramoyl-L-alanine amidase	YP_009201931.1	-
22	14825	15067	+	243	80	holin	YP_009197967.1	-
23	15201	15392	+	192	63	hypothetical protein	YP_009197968.1	-
24	15674	15784	+	111	36	hypothetical protein	YP_009197969.1	-
25	15781	16302	+	522	173	amidase domain-containing protein	YP_009197970.1	Potential reading frame shift.
26	16299	16487	+	189	62	amidase domain-containing protein	YP_009197970.1	
27	16523	17047	+	525	174	hypothetical protein	YP_009197971.1	-
28	17211	17318	+	108	35	hypothetical protein	YP_009197972.1	-
29	17380	18294	-	915	304	transposase	YP_009201939.1	-
30	18366	19019	-	654	217	helix-turn-helix transcriptional regulator	YP_009197974.1	-
31	19505	19708	-	204	67	hypothetical protein	YP_009197976.1	-
32	19710	20258	-	549	182	YolD-like family protein	YP_009197977.1	-
33	20500	20754	+	255	84	transposase	YP_009197978.1	-
34	20952	21641	-	690	229	transposase	YP_009197979.1	-

35	21647	21928	-	282	93	helix-turn-helix transcriptional regulator	YP_009197980.1	-
36	22638	23099	-	462	153	helix-turn-helix domain-containing protein	YP_009197981.1	-
37	23300	23557	+	258	85	hypothetical protein	YP_009201956.1	-
38	23703	23858	+	156	51	hypothetical protein	YP_009201956.1	-
39	23884	24624	+	741	246	antirepressor	YP_009197983.1	-
40	24651	24869	+	219	72	hypothetical protein	YP_009201958.1	-
41	24893	25162	+	270	89	hypothetical protein	YP_009201959.1	-
42	25159	25431	+	273	90	hypothetical protein	YP_009201960.1	-
43	25458	26216	+	759	252	antirepressor	YP_009201961.1	-
44	26213	26692	+	480	159	hypothetical protein	YP_009201962.1	-
45	26682	26930	+	249	82	hypothetical protein	YP_009201963.1	-
46	26935	27225	+	291	96	hypothetical protein	YP_009197985.1	-
47	27209	28741	+	1533	510	ATPase	YP_009197986.1	-
48	28728	28925	+	198	65	hypothetical protein	YP_009197987.1	-
49	28928	29830	+	903	300	recombinase RecT	YP_009197988.1	-
50	29843	30601	+	759	252	MBL fold metallo-hydrolase	YP_009197989.1	-
51	30598	30912	+	315	104	hypothetical protein	YP_008320387.1	-
52	30926	31756	+	831	276	hypothetical protein	YP_008320388.1	-
53	31722	33044	+	1323	440	DNA helicase	YP_009197992.1	-
54	33041	33352	+	312	103	hypothetical protein	YP_009201972.1	-
55	33345	34400	+	1056	351	DNA (cytosine-5-)-methyltransferase	YP_009201973.1	-
56	34387	34596	+	210	69	hypothetical protein	YP_009201974.1	-
57	34681	34851	-	171	56	hypothetical protein	-	-
58	34887	35477	+	591	196	resolvase	YP_009838694.1	-

59	35636	36094	+	459	152	hypothetical protein	YP_009201977.1	-
60	36145	36603	+	459	152	hypothetical protein	YP_009201978.1	-
61	36615	36896	+	282	93	hypothetical protein	YP_009201979.1	-
62	36835	38397	+	1563	520	integrase	YP_009201980.1	-
63	38537	38812	+	276	91	phosphomannomutase	YP_009201981.1	-
64	38841	39251	-	411	136	type II toxin-antitoxin system HicB family antitoxin	YP_009201982.1	-
65	39282	39518	-	237	78	type II toxin-antitoxin system HicA family toxin	YP_009201983.1	-
66	40494	40760	+	267	88	transglycosylase	YP_009201986.1	-
67	40757	41095	+	339	112	HNH endonuclease	YP_009201987.1	-

Table S4. Annotation table of phage 4/A

#	Location		Strand	Size		Probable function	Homologue	Comments
	Start	End		nt	aa			
1	1	297	+	297	98	terminase, small subunit	YP_009195191.1	-
2	278	2002	+	1725	574	terminase, large subunit	YP_008320338.1	-
3	1999	3252	+	1254	417	portal protein	YP_009203205.1	-
4	3236	3958	+	723	240	Clp, protease	YP_009203206.1	-
5	3955	5085	+	1131	376	major capsid protein	YP_008320341.1	-
6	5063	5236	+	174	57	hypothetical protein	YP_008320342.1	-
7	5229	5492	+	264	87	head-tail connector protein	YP_008320343.1	-
8	5489	5806	+	318	105	head closure protein	YP_008320344.1	-

9	5806	6234	+	429	142	hypothetical protein	YP_008320345.1	-
10	6218	6613	+	396	131	tail protein	YP_008320346.1	-
11	6615	7052	+	438	145	major tail protein	YP_008320347.1	-
12	7049	7387	+	339	112	hypothetical protein	YP_009195202.1	-
13	7414	7668	+	255	84	hypothetical protein	YP_008320349.1	-
14	7728	10364	+	2637	878	tail tape measure protein	YP_008320350.1	-
15	10361	11212	+	852	283	tail protein	YP_008320351.1	-
16	11215	12333	+	1119	372	*Siphovirus* ReqiPepy6 Gp37-like protein	YP_008320352.1	-
17	12330	13412	+	1083	360	tail protein	YP_009201927.1	-
18	13413	13760	+	348	115	hypothetical protein	YP_009201928.1	-
19	13757	13918	+	162	53	hypothetical protein	YP_009197964.1	-
20	13899	14138	+	240	79	hypothetical protein	YP_009197965.1	-
21	14135	14809	+	675	224	N-acetylmuramoyl-L-alanine amidase	YP_009201931.1	-
22	14819	15061	+	243	80	holin	YP_009197967.1	-
23	15195	15386	+	192	63	hypothetical protein	YP_009197968.1	-
24	15668	15778	+	111	36	hypothetical protein	YP_009197969.1	-
25	15775	16296	+	522	173	amidase domain-containing protein	YP_009197970.1	Potential reading frame shift.
26	16293	16481	+	189	62	amidase domain-containing protein	YP_009197970.1	
27	16517	17041	+	525	174	hypothetical protein	YP_009197971.1	-
28	17205	17312	+	108	35	hypothetical protein	YP_009197972.1	-
29	17374	18288	-	915	304	transposase	YP_009201939.1	-
30	18344	19012	-	669	222	helix-turn-helix domain-containing protein	YP_009197974.1	-
31	19498	19701	-	204	67	hypothetical protein	YP_009197976.1	-

32	19703	20251	-	549	182	YolD-like family protein	YP_009197977.1	-
33	20493	20747	+	255	84	transposase	YP_009197978.1	-
34	20945	21634	-	690	229	transposase	YP_009197979.1	-
35	21640	21921	-	282	93	helix-turn-helix transcriptional regulator	YP_009197980.1	-
36	22631	23092	-	462	153	helix-turn-helix domain-containing protein	YP_009197981.1	-
37	23293	23550	+	258	85	hypothetical protein	YP_009201956.1	-
38	23696	23851	+	156	51	hypothetical protein	YP_009201956.1	-
39	23877	24617	+	741	246	antirepressor	YP_009197983.1	-
40	24644	24862	+	219	72	hypothetical protein	YP_009201958.1	-
41	24886	25155	+	270	89	hypothetical protein	YP_009201959.1	-
42	25152	25424	+	273	90	hypothetical protein	YP_009201960.1	-
43	25451	26209	+	759	252	antirepressor	YP_009201961.1	-
44	26206	26685	+	480	159	hypothetical protein	YP_009201962.1	-
45	26675	26923	+	249	82	hypothetical protein	YP_009201963.1	-
46	26928	27218	+	291	96	hypothetical protein	YP_009197985.1	-
47	27202	28734	+	1533	510	ATPase	YP_009197986.1	-
48	28721	28918	+	198	65	hypothetical protein	YP_009197987.1	-
49	28921	29823	+	903	300	recombinase RecT	YP_009197988.1	-
50	29836	30594	+	759	252	MBL fold metallo-hydrolase	YP_009197989.1	-
51	30591	30905	+	315	104	hypothetical protein	YP_008320387.1	-
52	30919	31749	+	831	276	hypothetical protein	YP_008320388.1	-
53	31715	33037	+	1323	440	DNA helicase	YP_009197992.1	-
54	33034	33345	+	312	103	hypothetical protein	YP_009201972.1	-
55	33338	34393	+	1056	351	DNA (cytosine-5-)-methyltransferase	YP_009201973.1	-

56	34380	34589	+	210	69	hypothetical protein	YP_009201974.1	-
57	34674	34844	-	171	56	hypothetical protein	-	-
58	34880	35470	+	591	196	resolvase	YP_009838694.1	-
59	35629	36087	+	459	152	hypothetical protein	YP_009201977.1	-
60	36138	36596	+	459	152	hypothetical protein	YP_009201978.1	-
61	36608	36889	+	282	93	hypothetical protein	YP_009201979.1	-
62	36828	38390	+	1563	520	integrase	YP_009201980.1	-
63	38530	38805	+	276	91	phosphomannomutase	YP_009201981.1	-
64	38834	39244	-	411	136	type II toxin-antitoxin system HicB family antitoxin	YP_009201982.1	-
65	39275	39511	-	237	78	type II toxin-antitoxin system HicA family toxin	YP_009201983.1	-
66	40487	40753	+	267	88	transglycosylase	YP_009201986.1	-
67	40750	41031	+	282	93	HNH endonuclease	YP_009201987.1	-

Table S5. Annotation table of phage 5/A

#	Location		Strand	Size		Probable function	Homologue	Comments
	Start	End		nt	aa			
1	1	303	+	303	100	terminase, small subunit	YP_009195191.1	-
2	284	2008	+	1725	574	terminase, large subunit	YP_008320338.1	-
3	2005	3258	+	1254	417	portal protein	YP_009203205.1	-
4	3242	3964	+	723	240	Clp, protease	YP_009203206.1	-
5	3961	5091	+	1131	376	major capsid protein	YP_008320341.1	-

6	5069	5242	+	174	57	hypothetical protein	YP_008320342.1	-
7	5257	5427	+	171	56	hypothetical protein	YP_008320343.1	Potential reading frame shift.
8	5506	5823	+	318	105	head closure protein	YP_008320344.1	-
9	5823	6251	+	429	142	hypothetical protein	YP_008320345.1	-
10	6235	6630	+	396	131	tail protein	YP_008320346.1	-
11	6632	7069	+	438	145	major tail protein	YP_008320347.1	-
12	7066	7404	+	339	112	hypothetical protein	YP_009195202.1	-
13	7431	7685	+	255	84	hypothetical protein	YP_008320349.1	-
14	7745	10282	+	2538	845	hypothetical protein	YP_008320350.1	Potential reading frame shift.
15	10279	11130	+	852	283	tail protein	YP_008320351.1	-
16	11133	12251	+	1119	372	Siphovirus ReqiPepy6 Gp37-like protein	YP_008320352.1	-
17	12248	13330	+	1083	360	tail protein	YP_009201927.1	-
18	13331	13678	+	348	115	hypothetical protein	YP_009201928.1	-
19	13675	13836	+	162	53	hypothetical protein	YP_009197964.1	-
20	13817	14056	+	240	79	hypothetical protein	YP_009197965.1	-
21	14053	14727	+	675	224	N-acetylmuramoyl-L-alanine amidase	YP_009201931.1	-
22	14737	14979	+	243	80	holin	YP_009197967.1	-
23	15113	15304	+	192	63	hypothetical protein	YP_009197968.1	-
24	15586	15696	+	111	36	hypothetical protein	YP_009197969.1	-
25	15693	16214	+	522	173	amidase domain-containing protein	YP_009197970.1	-
26	16211	16399	+	189	62	amidase domain-containing protein	YP_009197970.1	-
27	16435	16959	+	525	174	hypothetical protein	YP_009197971.1	-
28	17123	17230	+	108	35	hypothetical protein	YP_009197972.1	-

29	17292	18011	-	720	239	transposase	YP_009201939.1	-
30	18182	18865	-	684	227	helix-turn-helix domain-containing protein	YP_009197974.1	-
31	19351	19554	-	204	67	hypothetical protein	YP_009197976.1	-
32	19556	20104	-	549	182	YolD-like family protein	YP_009197977.1	-
33	20346	20600	+	255	84	transposase	YP_009197978.1	-
34	20798	21487	-	690	229	transposase	YP_009197979.1	-
35	21493	21774	-	282	93	helix-turn-helix transcriptional regulator	YP_009197980.1	-
36	22484	22945	-	462	153	helix-turn-helix domain-containing protein	YP_009197981.1	-
37	23146	23403	+	258	85	hypothetical protein	YP_009201956.1	-
38	23549	23704	+	156	51	hypothetical protein	YP_009201956.1	-
39	23730	24470	+	741	246	antirepressor	YP_009197983.1	-
40	24497	24715	+	219	72	hypothetical protein	YP_009201958.1	-
41	24739	25008	+	270	89	hypothetical protein	YP_009201959.1	-
42	25005	25277	+	273	90	hypothetical protein	YP_009201960.1	-
43	25304	26062	+	759	252	antirepressor	YP_009201961.1	-
44	26059	26538	+	480	159	hypothetical protein	YP_009201962.1	-
45	26528	26776	+	249	82	hypothetical protein	YP_009201963.1	-
46	26781	27071	+	291	96	hypothetical protein	YP_009197985.1	-
47	27055	28587	+	1533	510	ATPase	YP_009197986.1	-
48	28574	28771	+	198	65	hypothetical protein	YP_009197987.1	-
49	28774	29676	+	903	300	recombinase RecT	YP_009197988.1	-
50	29689	30447	+	759	252	MBL fold metallo-hydrolase	YP_009197989.1	-
51	30444	30758	+	315	104	hypothetical protein	YP_008320387.1	-
52	30772	31602	+	831	276	hypothetical protein	YP_008320388.1	-
53	31568	32890	+	1323	440	DNA helicase	YP_009197992.1	-

54	32887	33198	+	312	103	hypothetical protein	YP_009201972.1	-
55	33191	34246	+	1056	351	DNA (cytosine-5-)-methyltransferase	YP_009201973.1	-
56	34233	34442	+	210	69	hypothetical protein	YP_009201974.1	-
57	34527	34697	-	171	56	hypothetical protein	-	-
58	34733	35323	+	591	196	resolvase	YP_009838694.1	-
59	35482	35940	+	459	152	hypothetical protein	YP_009201977.1	-
60	35991	36449	+	459	152	hypothetical protein	YP_009201978.1	-
61	36681	37061	+	381	126	integrase	YP_009201980.1	Potential reading frame shift.
62	37064	38347	+	1284	427	integrase	YP_009201980.1	
63	38487	38762	+	276	91	phosphomannomutase	YP_009201981.1	-
64	38791	39201	-	411	136	type II toxin-antitoxin system HicB family antitoxin	YP_009201982.1	-
65	39232	39468	-	237	78	type II toxin-antitoxin system HicA family toxin	YP_009201983.1	-
66	40444	40710	+	267	88	transglycosylase	YP_009201986.1	-
67	40707	41045	+	339	112	HNH endonuclease	YP_009201987.1	-
